# The learning curve of a novel seven-axis robot-assisted total hip arthroplasty system: a randomized controlled trial

**DOI:** 10.1186/s12891-024-07474-2

**Published:** 2024-04-30

**Authors:** Haocheng Sun, Hanpeng Lu, Qiang Xiao, Zichuan Ding, Zeyu Luo, Zongke Zhou

**Affiliations:** 1 Department of Orthopedics, West China Hospital, Orthopedic Research Institute, Sichuan University, Chengdu, China; 2 Department of Orthopedics, Chengdu Second People’s Hospital, Chengdu, China

**Keywords:** Robot assisted total hip arthroplasty, Learning curve, Cumulative summation, Prospective randomized controlled trial

## Abstract

**Bacground:**

The aim of this study was to assess the learning curve of a novel seven-axis robot-assisted total hip arthroplasty (RaTHA) system.

**Methods:**

A total of 59 patients who underwent unilateral total hip arthroplasty at our institution from June 2022 to September 2022 were prospectively included in the study. In this randomized controlled clinical trial, robot-assisted THA (RaTHA) and Conventional THA (CoTHA) were performed using cumulative sum (CUSUM) analysis to evaluate the learning curve of the RaTHA system. The demographic data, preopera1tive clinical data, duration of operation, postoperative Harris Hip Score (HHS), postoperative Western Ontario and McMaster Universities Arthritis Index (WOMAC) score, and duration of operation between the learning stage and the proficiency stage of the RaTHA group were compared between the two groups.

**Results:**

The average duration of operation of the RaTHA group was increased by 34.73 min compared with the CoTHA group (104.26 ± 19.33 vs. 69.53 ± 18.38 min, *p* < 0.01). The learning curve of the RaTHA system can be divided into learning stage and proficiency stage, and the former consists of the first 13 cases by CUSUM analysis. In the RaTHA group, the duration of operation decreased by 29.75 min in the proficiency stage compared to the learning stage (121.12 ± 12.84 vs.91.37 ± 12.92, *p* < 0.01).

**Conclusions:**

This study demonstrated that the surgical team required a learning curve of 13 cases to become proficient using the RaTHA system. The duration of operation, total blood loss, and drainage gradually shortened (decreased) with the learning curve stage, and the differences were statistically significant.

**Trial registration:**

Number: ChiCTR2200061630, Date: 29/06/2022.

## Introduction

Total hip arthroplasty (THA) is a well-established procedure designed to reduce pain, improve function and improve quality of life in patients with debilitating hip disease and has become one of the most commonly performed orthopedic intervention worldwide [1]. In fact, current research indicates that more than 600,000 people receive THA each year worldwide, and the number is expected to increase by approximately 1 million by 2030 [1, 2].

The success of traditional THA depends largely on the precision of implant placement, which requires extensive surgical experience [3]. Furthermore, with the widespread adoption of THA, the number of patients requiring hip revision due to dislocations, impingement, pelvic osteolysis, acetabular displacement, polyethylene wear, and unequal leg length is increasing, seriously affecting patient satisfaction and quality of life [4]. Compared with Conventional THA (CoTHA), several studies have shown that robot-assisted THA (RaTHA) can improve the accuracy of implant placement, achieve equal leg length, enhance hip offset, decreases in length of stay and costs, but may increase the readmissions and blood transfusions [5–9]. However, mastering RaTHA requires the surgeon to learn various techniques different from CoTHA, which can be time-consuming [10].

The learning curve of surgery may be closely related to surgical complexity [11]. A number of studies have reported the learning curve characteristics of RaTHA systems, but mainly retrospective studies, this study aims to address this gap by prospectively assessing the learning curve of a novel seven-axis machine-assisted THA system (Intelligent Technology Shenzhen Co., Ltd.).

## Materials and methods

Prior to patient enrollment, this prospective randomized controlled trial was registered on 29/06/2022 with the Chinese Clinical Trial Registry (ChiCTR2200061630). The data collected, analyzed, and reported in this trial were in accordance with the guidelines outlined in the Consolidated Standards of Reporting Trials (CONSORT) Statement.

### Participants

Between June 2022 and September 2022, a prospective randomized study involving 60 consecutive patients undergoing primary unilateral THA for Garden type I or II femoral neck fracture, Ficat stage III and IV femoral head necrosis, or hip osteoarthritis (Including hip dysplasia Crowe type I, type II) was conducted. The patients gave their written informed consent to inclusion in the trial before surgical intervention occurred. Exclusion criteria were as follows: (i) Active infection lesions in the hip joint or other parts of the body; (ii) Severe hip deformity and hip dysplasia with CROWE grade 3 or 4; (iii) Ankylosing spondylitis patients with bony ankylosis or severe stiffness; (iv) coagulation disorders; and (v) comorbidities such as uncontrolled hypertension, severe cardiovascular disorder and organ failure.

### Randomization, and blinding

The patients were randomly assigned to two groups: the RaTHA group (experimental group: 30 cases) and the CoTHA group (control group: 29 cases). Randomization was performed using a computer-generated list of random assignments. The patients, assessors of outcomes, and data collectors were unaware of their group assignments. Although the surgeon who conducted the RaTHA and CoTHA procedures was not blinded, they were not involved in data collection or postoperative management for this trial.

### Preoperative preparation

Patients in the RaTHA group underwent preoperative CT scans of bilateral hip and knee joints. The data was uploaded into the robotic system to build a 3D model for preoperative planning. Acetabular cup placement angles were set at an anteversion of 20° and an inclination of 40° relative to the functional pelvic plane (FPP) for all procedures (radiographic inclination of 42° and radiographic anteversion of 15°). All patients were required to take standard pelvic X-ray film preoperatively and the requirements were as follows: the X-ray projection center was at the midpoint of the bilateral hip joints, the legs were straight, and the internal rotation was 15–20º. Two surgeons independently performed template measurements on plain radiographs before operation, and performed preoperative planning to select the appropriate prosthesis (acetabular cup, femoral head and femoral stem) to improve bone contact and achieve equal leg length and offset. During the procedure, the acetabular cup position is determined by using mechanical navigation.

### Surgical technique

All surgical procedures were performed by the same specialist surgical team which had no previous experience with robot-assisted systems for THA. During the procedure the patients were placed under general anesthesia, lying on their sides on the operating table. All THA was performed through posterolateral approach. All RaTHA procedures were performed using a single robot-assisted surgical system (YUANHUA-THA; Yuanhua Orthopaedic Robotics Limited, Shenzhen, China), which was a semiactive surgical robotic designed to assist for patients undergoing THA. During the operation, the surgeon can register the preoperative image with the real bone surface, adjust the position of the prosthesis accurately, and finally determine the surgical plan, through the system. The surgical procedures were described in the technical manuals provided by the manufacturer.

In the RaTHA group, the camera stand and robotic arm were placed in a suitable position beside the patient’s head and on the opposite side of the main surgeon, operating according to the system. Then, the surgeon positioned the femur, placed the proximal femoral check nail vertically at the top of the greater trochanter of the femur, while placing the distal femoral electrode sheet at the inferior pole of the patella, and then the surgeon calibrated the three-dimensional spatial position of the femur by inputting the marking points at both ends of the femur with a probe. During the procedure, the pelvic tracker, a pin was placed 2 cm above the anterior superior iliac spine along the direction of the iliac flanks. After the surgeon has aligned the femur, the robot guides the surgeon through the femoral neck osteotomy with a probe to determine the position of the osteotomy surface, which usually is 1 cm above the lesser trochanter, and the retained length of the femoral neck was measured, marked, and osteotomized according to preoperative planning. After adequate exposure of the acetabulum, the acetabular inspection nail is next inserted, and the surgeon registered the acetabulum by a probe, selecting four points on the anterior superior, superior external, posterior inferior and anterior inferior of the acetabulum. After the initial approximation of the position of the acetabulum, 15 points on the inner surface of the acetabulum and 15 points on the outer surface of the acetabulum are registered. If the deviation is less than 0.1 cm and 1degree, it indicates that acetabular registration is successful. The acetabulum was rasped and filed with a mechanical arm under the limits of the defined rasped and filed with a mechanical arm within the defined preoperatively planned limits of inclination and anteversion. After filing, the appropriate volume of bone and soft tissue was removed as planned, and then the acetabular component was fitted with the assistance of the robotic arm which limited the inclination and anteversion. The depth of cup placement was determined by the depth of the acetabular grinding file. Afterwards the surgeon selected five points in the plane of the cup by means of a probe to confirm the anterior inclination and abduction angle of the cup. After the position of the acetabular component is confirmed, the femoral component was manually implanted and after the hip reduction, the surgeon checked the off-set, limb length, and joint stability.

In the CoTHA group, femoral neck osteotomy was performed for the implantation of prosthesis according to preoperative template measurement. No screws were placed in any of the patients. All patients underwent the cementless prosthesis (ceramics on ceramic interface) came from Zhengtian Medical Instrument (Tianjin, China). Drain was placed in both groups.

### Clinical outcome measures

The primary outcome was duration of operation. Secondary outcomes included total blood loss (TBL), drainage, function recovery, and postoperative complications.

Duration of operation was defined as the time from the start of skin incision to the end of incision closure. Postoperative TBL was assessed by calculating the pre- and postoperative blood volumes. The hemoglobin (Hb) concentration and hematocrit (Hct) were measured preoperatively and on postoperative day (POD) 1. Estimated total blood loss was calculated as described by Gross and Nadler et al. [12, 13] from the change in Hct. Drainage was measured by a graduated cylinder at 24 h postoperatively.

### Functional assessments

The assessment of hip function was conducted utilizing the Harris Hip Score (HHS) and the Western Ontario and McMaster Universities Arthritis Index (WOMAC) techniques for evaluating osteoarthritis.

### Harris hip score

The Harris Hip Score (HHS) is a comprehensive set of numerical rating criteria developed by Harris and widely utilized for evaluating hip joint function. The assessment encompassed aspects such as pain, function, range of motion, and deformity. The HHS score has a maximum possible value of 100, and scores between 90 and 100 are considered excellent, scores between 80 and 89 are rated as good, scores between 70 and 79 are rated as fair, while scores below 70 are classified as poor [14–16].

### WOMAC score

The scale evaluates the structure and function of the joint based on 24 items across three domains: pain, stiffness, and function. Elevated WOMAC scores are indicative of more severe arthritis. Scores below 80 suggest mild arthritis, scores between 80 and 120 indicate moderate arthritis, while scores exceeding 120 signify severe arthritis [15, 17]. .

### Learning curve analysis

The term learning curve (LC) describes the trend of surgical proficiency, which is closely related with personal experience and surgical complexity [10, 11, 18–20], and LC was analyzed by cumulative summation (CUSUM) [21, 22]. CUSUM was used to sort all cases according to the operation date, and the deviation between the observed value of each sample and the target value (the mean duration of operation in all cases in the robot-assisted THA group) was calculated. The CUSUM value was a cumulative mixture of increments with each failure and decrements with each success. Its results were presented in a chart with surgical sequence on the x-axis and the corresponding CUSUM value on the y-axis. Progressive curve fitting was used on the scatter plots obtained by CUSUM. The end of the learning curve was determined as the point where the slope of the curve changed from positive to negative [22–26]. Considering CUSUM analysis, the learning stage was established by the number of cases in the learning curve and the proficiency stage was represented by all subsequent cases in the study.

### Statistical analysis

Descriptive statistics of patient demographics and surgical data were compared separately between groups and between stages to ensure that learning curves were not affected by any inherent differences in patient characteristics. In the end, the duration of operation, TBL, drainage, functional score, and postoperative complications were compared across different stages.

Statistical analysis was performed using SPSS (version 22.0; IBM). Continuous variables are reported as means and standard deviations (SD) and compared between groups using independent-samples t-tests. Categorical variables were expressed as frequencies and compared using Pearson’s chi-square test or Fisher’s exact test when appropriate. A p-value of < 0.05 was considered statistically significant.

## Results

### Patient characters

**Fig. 1:**
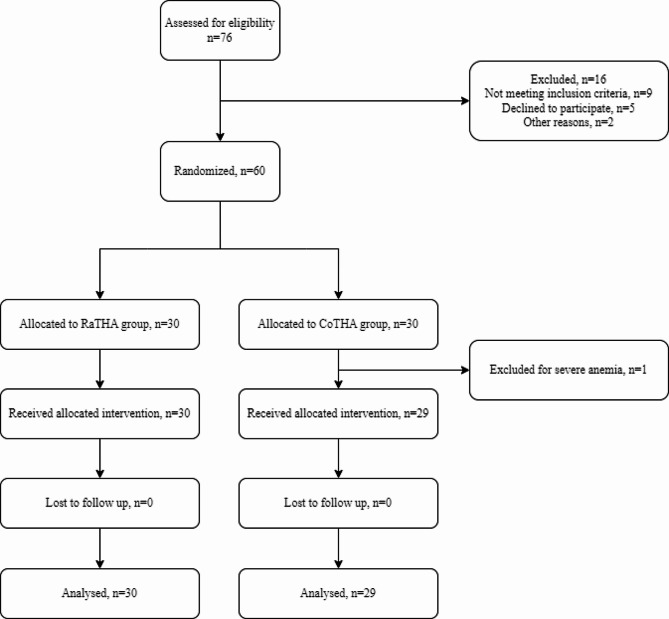
Consolidated Standards of Reporting Trials (CONSORT) flowchart of the study

From June 2022 to September 2022, a total of 76 patients who underwent primary unilateral THA were initially assessed for eligibility. Among them, six patients were excluded from the study: four patients did not meet the inclusion criteria, and two patients declined to participate. Subsequently, 60 patients were randomly assigned to two groups, with 30 patients in each group. However, one patient in the control group was later excluded due to severe anemia (as shown in Fig. 1). Ultimately, a total of 59 patients received the assigned intervention. No significant differences were observed between the two groups in terms of baseline demographic variables and perioperative characteristics, as indicated in Table 1.

**Table 1:** Baseline of the patients

	RaTHA (*n* = 30)	CoTHA (*n* = 29)	*P* value
Age (year)	56.10 ± 12.29	56.57 ± 11.74	0.88
Sex, (no. of patients [%])			
Male	13(43.33)	9(31.03)	0.33
Operative side, (no. of patients [%])			
Left	16(53.33)	15(51.72)	0.90
Diagnosis			
Osteonecrosis of femoral	10	6	0.21
Osteoarthritis	19	24	
Height (m)	1.60	1.59	0.50
Weight (kg)	61.83	58.58	0.28
BMI (kg/m^2^)	24.28 ± 2.89	23.10 ± 3.38	0.15
HHS	53.55 ± 13.93	54.71 ± 9.52	0.71
WOMAC	47.12 ± 13.71	45.91 ± 10.54	0.89

RaTHA, robot-assisted total hip arthroplasty; CoTHA, conventional total hip arthroplasty; BMI, Body Mass Index; HHS, Harris Hip Score; WOMAC, Western Ontario and McMaster Universities Arthritis Index

### Primary outcome

**Fig. 2:**
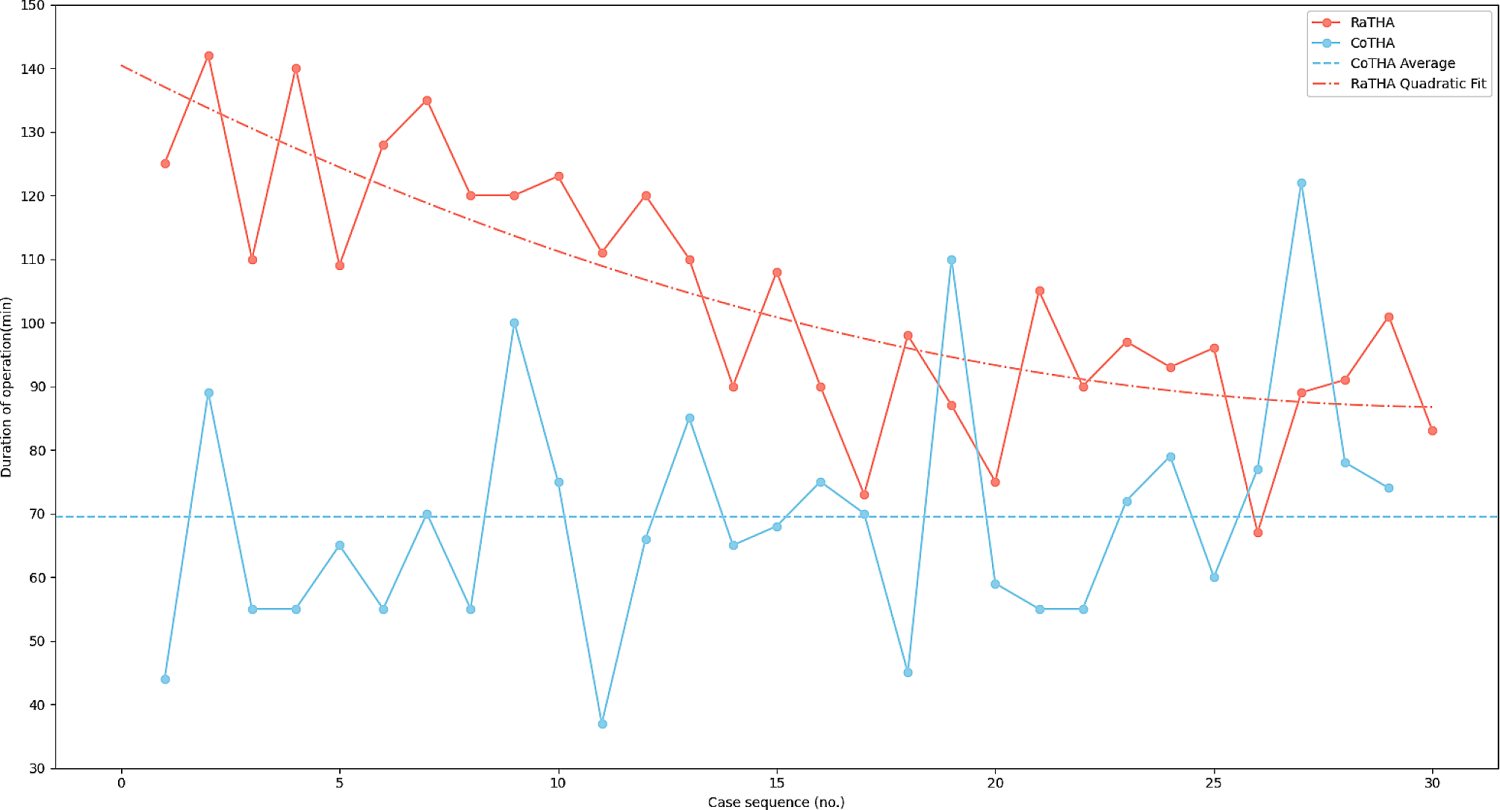
The duration of operation for the 59 patients undergoing robot-assisted THA in chronological order

The average duration of operation of the RaTHA group (104.26 ± 19.33 min, ranging from 67 to 142 min) was significantly increased 34.73 min than that of the CoTHA group (69.53 ± 18.38 min, ranging from 37 to 110 min) (*P* < 0.01). And the duration of operation of the RaTHA group showed a downward trend as a whole (Fig. 2).

### CUSUM

**Fig. 3:**
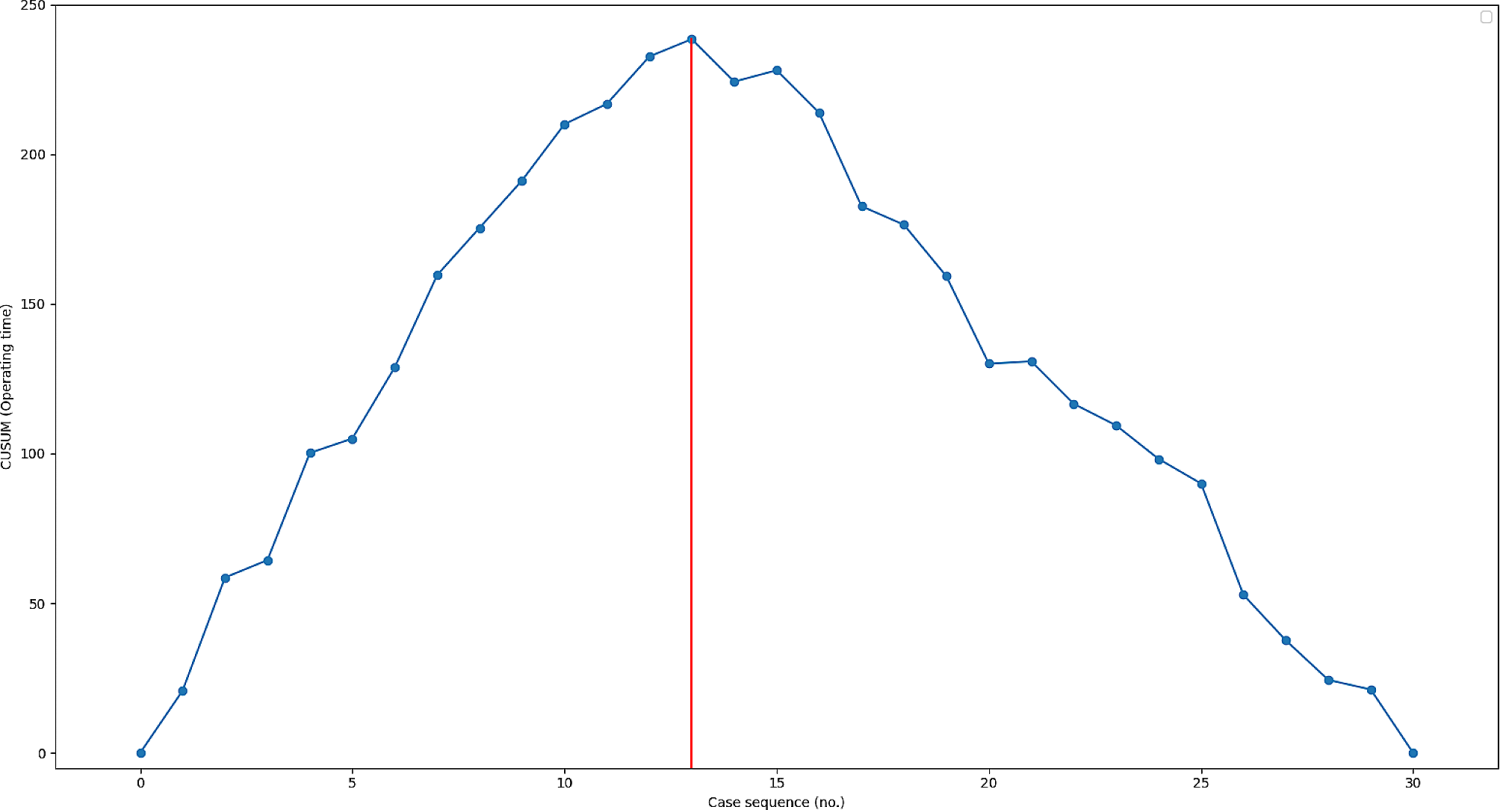
CUSUM learning curve

According to the results of CUSUM analysis, the thirteenth patient in the RaTHA group began to have an inflection point in the CUSUM curve. Then the RaTHA group was divided into two stages, the learning stage (the first 13 cases) and the proficiency stage (cases 14–30) (Fig. 3). Demographic characteristics were basically similar between the two stages (Table 2).

**Table 2:** Baseline between learning stage and proficiency stage

	Learning Stage (*n* = 13)	Proficiency Stage (*n* = 17)	*P* value
Age (year)	54.50 ± 7.56	58.63 ± 9.50	0.40
Sex, (no. of patients [%])			
Male	8(61.5)	5(29.41)	0.08
operative side, (no. of patients [%])			
Left	8(61.54)	8(47.06)	0.43
Diagnosis			
Osteonecrosis of femoral	4	6	0.71
Osteoarthritis	9	10	
Height (m)	1.61 ± 0.08	1.63 ± 0.09	0.59
Weight (kg)	57.42 ± 6.34	64.63 ± 8.96	0.12
BMI (kg/m^2^)	22.25 ± 1.35	24.20 ± 1.83	0.05
HHS	57.60 ± 15.75	50.11 ± 12.23	0.16
WOMAC	43.57 ± 15.71	50.40 ± 11.67	0.19

BMI, Body Mass Index; HHS, Harris Hip Score; WOMAC, Western Ontario and McMaster Universities Arthritis Index

### Clinical and functional outcomes

There was no significant difference in the TBL, drainage, postoperative HHS and WOMAC scores between the RaTHA and CoTHA groups (*P* > 0.05) (Table 3) In the RaTHA group, there was no significant difference in postoperative HHS and WOMAC scores between patients at different learning curve stages (*P* > 0.05) (Table 3). With the progress of the learning curve, the duration of operation changed from 121.1 ± 12.8 min decreased to 91.3 ± 12.9 min (*p* < 0.01), TBL decreased from 1616.6 ± 302.0 ml to 1023.1 ± 255.4 ml (*p* < 0.01), drainage volume decreased from 220.0 ± 114.1 ml to 127.0 ± 82.3 ml (*p* = 0.02 < 0.05) (Table 3).

**Table 3:** Clinical outcomes of RaTHA and CoTHA

	RaTHA	CoTHA	*P* value	Learning Stage	Proficiency Stage	*P* value
Duration of operation (min)	104.26 ± 19.33	69.53 ± 18.38	< 0.01	121.12 ± 12.84	91.37 ± 12.92	< 0.01
TBL (ml)	1280.33 ± 397.21	1105.22 ± 476.34	0.89	1616.61 ± 302.04	1023.12 ± 255.43	< 0.01
Drainage (ml)	167.92 ± 106.41	160.03 ± 113.44	0.29	220.03 ± 114.12	127.01 ± 82.34	0.02
HHS	96.81 ± 5.15	97.23 ± 4.26	0.73	96.22 ± 5.67	97.11 ± 4.96	0.60
WOMAC	4.36 ± 4.54	4.08 ± 4.27	0.88	3.54 ± 4.05	5.18 ± 4.92	0.34

RaTHA, robot-assisted total hip arthroplasty; CoTHA, conventional total hip arthroplasty; TBL, total blood loss; HHS, Harris Hip Score; WOMAC, Western Ontario and McMaster Universities Arthritis Index

### Efficacy outcomes

After surgery, the anteversion angle of the cup in the RaTHA group measured 20.8 ± 4.2°, compared to 18.7 ± 5.6° in the CoTHA group (*p* = 0.11), and the abduction angle was 36.4 ± 3.5°, compared to 37.6 ± 5.8° in the CoTHA group (*p* = 0.34). These measurements did not show a statistically significant difference, as presented in Table 4. Moreover, the rate of cup orientation within in the safe zone of Lewinnek et al. [27] was 96.7% (29/30) in the RaTHA group, which did not show a statistically significant difference from the 82.8% (24/29) observed in the CoTHA group (*p* = 0.08). Similarly, the leg length difference in the RaTHA group was 4.1 ± 3.6 mm, compared to that in the CoTHA group, which was 5.1 ± 4.4 mm, did not show significantly difference.

**Table 4:** Efficacy outcomes of RaTHA and CoTHA

	RaTHA (*n* = 30)	CoTHA (*n* = 29)	*P* value
Anteversion (°)	20.8 ± 4.2	18.7 ± 5.6	0.11
Inclination (°)	36.4 ± 3.5	37.6 ± 5.8	0.34
Lewinnek Safe zones, (no. of patients [%])	29/30 (96.67)	24/29 (82.76)	0.08
Leg length difference (mm)	4.1 ± 3.6	5.1 ± 4.4	0.34

RaTHA, robot-assisted total hip arthroplasty; CoTHA, coventional total hip arthroplasty; TBL, total blood loss

### Complications

In the postoperative period, all incisions exhibited early healing, and patients experienced a prompt and satisfactory recovery of hip function without encountering complications such as hip dislocation, aseptic loosening, or periprosthetic infection. Throughout the follow-up period, no complications were observed, including periprosthetic fracture, hip dislocation, aseptic loosening, or periprosthetic infection.

## Discussion

This study is the first prospective randomized controlled clinical study of the learning curve of a novel seven-axis robot-assisted total hip arthroplasty (RaTHA) system. The primary finding of our research suggests that surgeons may experience a learning curve of around 13 cases in order to achieve proficiency with this novel RaTHA system. Despite the presence of this learning curve, we did not observe any statistically significant differences in the clinical and functional assessments between the robot-assisted THA group and the conventional THA group.

The CUSUM method is presently widely recognized as a reliable approach for analyzing the inflection point of a learning curve, based on the duration of the operation [23, 28]. Many previous studies have evaluated the learning curves of robot-assisted THA systems [28–30]. In comparison to conventional THA, which necessitated up to 50 cases for surgeons to achieve optimal cup positioning, the learning curve for robot-assisted surgery was considerably shorter, with 13 attempts. Redmond [2] reported in a previous study that a learning curve for robot-assisted THA was observed, with a decrease in the duration of operation after 35 cases, which was notably longer than our findings. Consistent with our study’s findings, another report focusing on the learning curve of MAKO robot-assisted THA revealed that the duration of the operation stabilized after the first 14 cases [31]. Additionally, a cohort study [23] demonstrated that robot-assisted THA exhibited a learning curve of 14 cases in terms of the operation duration. These observations further support the results obtained in our present study.

The duration of operation is influenced by a number of factors, not only related to the operator’s familiarity with the procedure, but also could be decided by the complexity of the case, the scheduling of the surgery, and the operator’s energy of the time. In this study, we used parallel-control, with the same conditions in the experimental and control groups and synchronized study times to minimize biasing factors as possible. All surgeries were performed by a same surgical team, and we included patients with conditions within a narrow range: (Garden type I or II femoral neck fracture, Ficat stage III and IV femoral head necrosis, or hip dysplasia Crowe type I and II) to reduce the bias due to complexity of the case. During the period of this clinical trial, we also randomized the daily surgical schedule to reduce the bias due to the number of rounds of the surgical schedule.

The results of this study indicated that there was no significant difference in TBL and drainage between the RaTHA and CoTHA groups. Prolonged surgical procedures were correlated with a heightened susceptibility to blood loss and surgical trauma. Conversely, a shorter surgical duration would mitigate these risks. In RaTHA group, the duration of operation, TBL, and drainage gradually decreased as the surgeon’s learning curve progressed. With the operator’s proficiency in surgical instruments and the optimization of surgical procedures, the time for total hip arthroplasty under the assistance of the robotic arm can be close to that of conventional surgery in the later proficiency stage. In the proficiency stage the duration of operation is significantly reduced.

Bukowski et al. [32] reported clinical outcomes of robot-assisted THA with a minimum one-year follow-up and found that patients who underwent robot-assisted THA had superior clinical outcomes compared to a manual group. However, it is worth noting that there is currently a lack of large multicenter studies assessing clinical outcomes after robot-assisted THA. In the present study, we did not observe a significant increase in the Harris Hip Score (HHS) and Western Ontario and McMaster Universities Osteoarthritis Index (WOMAC) for patients who underwent robotic arm-assisted THA compared to experienced surgeons using manual techniques.

In addition, it is important to note that our study did not identify any significant differences in postoperative complications within our sample. However, it is crucial to acknowledge that drawing definitive conclusions about the impact of adopting RaTHA on postoperative outcomes is challenging due to several factors. Firstly, our sample size was relatively small, which may have limited our ability to detect rare events and observe significant differences. Furthermore, our analysis only considered complications up to 6 months postoperatively, potentially overlooking longer-term effects. Nevertheless, our findings align with existing literature reports that have similarly found no significant disparity in postoperative complication rates between the learning and proficiency stages following the adoption of RaTHA systems.

This study had certain limitations that should be acknowledged. Firstly, the surgeon and data collectors were not blinded to the group assignment. However, it is important to note that patients, anesthesiologists, and outcome assessors were blinded, and the surgeon did not participate in the data collection. Secondly, it is worth mentioning that this study was conducted in a single center, with all operations performed by the same proficient surgeon who had completed over 3000 THAs. Consequently, it remains uncertain whether the surgical technique employed may influence the study’s outcomes, and the learning curve could potentially vary for surgeons with lower THA volumes or less experience in THA. The safe zone established by Lewinnek et al. [27] is the most widely used range of acceptable angles with inclination of 30° to 50° and anteversion of 5° to 25°, and was used in our study to regard the safety of the robot-assisted THA. In our study, 96.7% (29/30) of cups in the RaTHA group, were placed in the safe zone of Lewinnek et al. compared with 82.76% (24/29) using the manual technique (*p* = 0.34), which demonstrated that the safety of robot-assisted THA. However, we did not assess femoral anteversion, while we took in to account the length of legs, which showed that there was no significant difference between RaTHA group and CoTHA group. And this is one of the limitations of our study.

## Conclusion

The surgical team required a learning curve of 13 cases to become proficient using the RaTHA system. The duration of operation, TBL, and drainage gradually shortened (decreased) with the learning curve stage, and the differences were statistically significant (*P* < 0.05).

## Data Availability

The datasets generated and analyzed during the current study are not publicly available but are available from the corresponding author on reasonable request.

## Abbreviations

RaTHA: Robot-assisted total hip arthroplasty
CoTHA: conventional total hip arthroplasty
CUSUM: Cumulative sum
BMI: Body mass index
HHS: Harris hip score
WOMACL: Western Ontario and McMaster universities arthritis index
TBL: Total blood loss
